# Leishmaniasis in Germany

**DOI:** 10.3201/eid0907.030023

**Published:** 2003-07

**Authors:** Gundel Harms, Gabriele Schönian, Hermann Feldmeier

**Affiliations:** * Charité, Humboldt University Berlin, Berlin, Germany; † Free University of Berlin, Berlin, Germany

**Keywords:** Leishmaniasis, Germany, risk factors, HIV infection, dispatch

## Abstract

In 2000, a reference center was created to systematically record leishmaniases in Germany. We analyzed 58 cases of leishmaniases imported during a 2-year period. These findings will serve as a baseline for the sandfly vector’s anticipated northward move because of global warming and as an advisory for immunocompromised persons traveling to leishmaniasis-endemic areas.

Leishmaniases compose a spectrum of protozoal diseases currently endemic in 88 countries in Asia, Africa, the Americas, and southern Europe. The geographic distribution of leishmaniases has widened, and the disease is reported in areas in which leishmaniasis was previously nonendemic (*1*). Apart from cutaneous, mucocutaneous/mucosal, and visceral leishmaniases, HIV-1–associated leishmaniasis acquired in southern Europe and in other parts of the world have been observed in increasing numbers (*1*,*2*).

Leishmaniasis is not notifiable in Germany. In September 2000, a national advice and reference center was created at the Institute of Tropical Medicine in Berlin; the aim of the center was to monitor the frequency, origin, and type of leishmaniases seen in Germany; to advise physicians; and to improve information for travelers to disease-endemic areas. The healthcare professionals were informed about the reference center by the Robert-Koch-Institute*,* the center for surveillance of infectious diseases in Germany, as well as through the Journal of the German Medical Association, which is received by every registered physician (*3*). Leishmaniasis was diagnosed if parasites were detected in smears, culture, histologic sections, by polymerase chain reaction (PCR) of lesion biopsy specimens, bone marrow, or peripheral blood. For detection of *Leishmania*-specific antigen the small subunit (ssu rRNA), the internal transcribed spacer (ITS-1) region of the ribosomal RNA genes, or both, were amplified by PCR (*4*). *Leishmania* complexes and species were determined by digestion of the ribosomal ITS-1 PCR product with restriction enzymes (*4*).

Within 2 years, 70 cases of leishmaniases (43 cutaneous or mucocutaneous/mucosal; 27 visceral) were reported. For 58 case-patients (35 cutaneous or mucocutaneous/mucosal; 23 visceral), data were available on the age, sex, residence, travel destination, possible exposure location, reason for travel, duration of stay, duration and type of symptoms, concomitant diseases or therapies, type of diagnosis, and treatment received.

## Cutaneous and Mucosal Leishmaniasis

Of the 35 patients with cutaneous or mucocutaneous/mucosal leishmaniasis, 30 were German tourists (Table 1). The male-to-female ratio was 1.5:1. Ten had contracted cutaneous or mucocutaneous/mucosal leishmaniasis in Europe, 11 in Central and South America, 6 in Asia, and 3 in Africa. Two persons had been infected during work stays of 1 to 4 months in French Guyana, one each in Peru and Libya, and one patient had immigrated from Afghanistan.

**Table 1:** Characteristics of patients with cutaneous and mucosal leishmaniasis, Germany^a^

No.	Exposure	Status	Sex	Age (y)	*Leishmania* species	Treatment	Outcome
Europe							
1	France	Tourist	F	64	*L. donovani* complex	No treatment	No cure
2	Italy	Tourist	F	24	*L. donovani* complex	Liposomal amphotericin B	Improved
3	Malta	Tourist	M	22	*L. donovan*i complex	Perilesional pentavalent antimonials	Cured
4	Malta	Tourist	M	39	*L. donovan*i complex	Perilesional pentavalent antimonials	Cured
5	Malta	Tourist	F	57	n.d.	Perilesional pentavalent antimonials	Cured
6	Malta	Tourist	M	62	n.d.	Perilesional pentavalent antimonials	Cured
7	Spain (Majorca)	Tourist	F	5	n.d.	Pentamidine isethionate	Cured
8	Spain (Majorca)	Tourist	M	13	*L. donovan*i complex	Perilesional pentavalent antimonials	Cured
9	Spain	Tourist	F	31	*L. donovani* complex	No treatment	Unknown
10	Spain	Tourist	M	33	*L. donovan*i complex	Antibiotic	Cured
Americas							
11	Belize	Tourist	M	36	*L. braziliensis* complex	Liposomal amphotericin B	Unknown
12	Belize	Tourist	F	32	n.d.	IFN-gamma	Cured
13	Bolivia	Tourist	M	35	*L. braziliensis* complex	Liposomal amphotericin B	Unknown
14	Brazil	Tourist	M	25	n.d.	Systemic pentavalent antimonials	Cured
15	Brazil	Tourist	M	39	*L. braziliensis* complex	Liposomal amphotericin B	Unknown
16	Brazil	Tourist	M	33	*L. braziliensis* complex	Liposomal amphotericin B	Cured
17	Ecuador	Tourist	F	29	*L. braziliensis* complex	Liposomal amphotericin B	Cured
18	Ecuador	Tourist	M	36	n.d.	Liposomal amphotericin B	Cured
19	French Guyana	Work stay	M	28	*L. braziliensis* complex	Liposomal amphotericin B	Cured
20	French Guyana	Work stay	M	22	*L. braziliensis* complex	Liposomal amphotericin B	Cured
21	Guatemala	Tourist	M	31	*L. mexicana*	Ketokonazole	Cured
22	Peru	Work stay	F	25	*L. braziliensis* complex	Liposomal amphotericin B	Cured
23	Peru	Tourist	F	33	*L. braziliensis* complex	Liposomal amphotericin B	Cured
24	Peru	Tourist	M	35	*L. braziliensis* complex	Liposomal amphotericin B	Cured
Asia							
25	Afghanistan	Immigrant	M	21	*L. tropica*	Aminosidine ointment	No cure
26	Afghanistan	Tourist	F	12	n.d.	Aminosidine ointment	Cured
27	United Arab Emirates	Tourist	F	44	n.d.	Perilesional pentavalent antimonials	Cured
28	Syria	Tourist	M	5	n.d.	Perilesional pentavalent antimonials	Unknown
29	Syria	Tourist	F	3	n.d.	Perilesional pentavalent antimonials	Unknown
30	Turkey	Tourist	F	33	n.d.	Antibiotic	Cured
31	Turkey	Tourist	M	37	*L. donovani* complex	Liposomal amphotericin B	Improved
Africa							
32	Egypt	Tourist	M	25	*L. tropica*	Aminosidine ointment	No cure
33	Egypt	Tourist	F	27	*L. tropica*	Aminosidine ointment	Cured
34	Kenya	Tourist	M	50	n.d.	Perilesional pentavalent antimonials	Cured
35	Lybia	Work stay	M	34	n.d.	Aminosidine ointment	Cured

^a^n.d., not done; IFN, interferon.

The median duration of lesions until the diagnosis of leishmaniasis was made was 4 months (range 3 weeks to 2 years). Sixteen patients had more than one lesion (median 2, range 1–6 lesions). Seventeen lesions were located in the face, including mouth and nose, 28 on the upper extremities and 21 on the lower extremities. Lesions were ulcerated in 39 cases, papular-nodular in 24, and plaque-like in 3. Parasites were detected in 13 of 20 smears, in 9 of 10 cultures, and in 14 of 16 histologic sections; by using PCR, *Leishmania-*specific DNA was detected in 16 of 16 biopsy specimens.

Patient 1 had lesions in the mouth caused by *L. infantum*. She was under continuous immunosuppressive treatment for severe bronchial asthma. Patient 2 had received methotrexate and steroids for treatment of systemic collagenosis for several weeks. Both patients were tested for leishmanial infection in the blood; in both patients, the *Leishmania*-specific PCR of the buffy coat of the blood was positive. Patient 22 had mucocutaneous leishmaniasis of the nasal septum. She had been treated for a skin lesion caused by *L. braziliensis* 3 years earlier.

## Visceral Leishmaniasis

A total of 18 of the 23 visceral leishmaniasis patients were German tourists; 3 were immigrants from Angola, Iran, and Togo; and 2 were visitors from Italy and Portugal (Table 2). The male-to-female ratio was 6.7:1.

**Table 2:** Characteristics of patients with visceral leishmaniasis, Germany^a^

No.	Exposure	Status	Sex	Age	Risk factor	Leishmania species	Treatment	Outcome
1	Italy	Tourist	M	2 y	Child	n.d.	Liposomal amphotericin B	Cured
2	Italy	Tourist	F	5 y	Child	n.d.	Liposomal amphotericin B	Cured
3	Italy	Tourist	M	11 y	Child	n.d.	Liposomal amphotericin B	Cured
4	Spain	Tourist	F	8 mo	Child	n.d.	Liposomal amphotericin B	Cured
5	Spain	Tourist	M	9 mo	Child	*L. donovani* complex	Liposomal amphotericin B	Cured
6	Iran	Immigrant	M	7 y	Child	*L. donovan*i complex	Liposomal amphotericin B	Cured
7	Spain	Tourist	M	43 y	HIV	*L. donovan*i complex	Liposomal amphotericin B; Maintenance therapy: HAART plus liposomal amphotericin B once monthly	No relapse for 6 months
8	Spain (Ibiza)	Tourist	M	48 y	HIV	*L. donovani* complex	Liposomal amphotericin B; Maintenance therapy: HAART plus liposomal amphotericin B once monthly	No relapse for 8 months
9	Portugal	Visitor	M	29 y	HIV	n.d.	Liposomal amphotericin B; Maintenance therapy: HAART plus liposomal amphotericin B once monthly	unknown
10	France	Tourist	M	31y	HIV	*L. donovan*i complex	Liposomal amphotericin B; Maintenance therapy: HAART plus liposomal amphotericin B once monthly	Relapse after 4 months; retreatment with Liposomal Amphotericin B; No relapse for 3 months
11	Angola	Immigrant	M	40 y	HIV	*L. donovani* complex	Systemic pentavalent antimonials Maintenance therapy: HAART plus pentavalent antimonials once monthly	unknown
12	Togo	Immigrant	M	37 y	HIV	*L. donovani* complex	Liposomal amphotericin B	Cured
13	Italy (Sicily)	Visitor	M	31 y	Thymoma	n.d.	Liposomal amphotericin B	Cured
14	Italy (Ischia)	Tourist	M	67 y	Methotrexate/steroids	*L. donovani* complex	Liposomal amphotericin B	Cured
15	Italy (Sicily)	Tourist	M	68 y	Methotrexate	n.d.	Liposomal amphotericin B	Cured
16	Italy (Ischia)	Tourist	M	70 y	Splenectomy	n.d.	Liposomal amphotericin B	Cured
17	Spain	Tourist	M	51 y	Splenectomy	*L. donovani* complex	Liposomal amphotericin B	Cured
18	Greece (Korfu)	Tourist	M	66 y	Diabetes mellitus Hypertonus	n.d.	Liposomal amphotericin B	Cured
19	Spain	Tourist	M	52 y	Hypertonus Hypercholesterolemia	n.d.	Liposomal amphotericin B	Cured
20	Greece (Korfu)	Tourist	M	45 y	Diabetes mellitus Emphysema	*L. donovani* complex	Systemic pentavalent antimonoials	Cured
21	Tunisia	Tourist	M	53 y	Hypertonus	*L. donovani* complex	Liposomal amphotericin B	Cured
22	Malta	Tourist	F	55 y	-	n.d.	Liposomal amphotericin B	Cured
23	China	Tourist	M	67 y	-	n.d.	Liposomal amphotericin B	Cured

^a^n.d., not done; IFN, interferon.

The median time between symptom onset and the correct diagnosis was 4 months (range 1–16 months). All case-patients had fever, 17 (74%) had splenomegaly, 11 (48%) hepatomegaly, 20 (87%) anemia, 17 (74%) leukopenia, and 8 (35%) thrombocytopenia.

Bone marrow smears indicated *Leishmania* in 18 of 20, bone marrow culture in 6 of 7, bone marrow histologic sections in 7 of 8, PCR of the bone marrow in 8 of 9, and PCR of the buffy coat of the blood in 7 of 7 cases. Additionally, antibodies were detected in medium to high concentration by an immunofluorescence test, enzyme-linked immunosorbent assay (ELISA), or both, in 14 of 15 cases. Species was identified in 7 of 18 visceral cases contracted in southern Europe and indicated *Leishmania* belonging to the *L. donovani* complex, which implicated infection with *L. infantum*.

Six cases of visceral leishmaniasis occurred in children 2 months of age to 11 years of age. Four German tourists and two immigrants had long-known HIV infection (median duration 3 years, range 8 months–6 years). All HIV–co-infected patients had CD4-cell counts below 200/μL (median 108, range 23–185 CD4 cells/μL) when the diagnosis of visceral leishmaniasis was made. Of the remaining 11 patients, 1 had a thymoma with impaired T-helper-1 cell function, 2 had received intermittent immunosuppressive therapy (methotrexate and steroids) for rheumatologic disease, and 2 patients had their spleens removed. Three patients were in an impaired general condition because of combinations of diabetes, hypertonus, hypercholesterolemia, and emphysema. In the remaining three patients (53–68 years of age), apart from hypertonus in one, no impairing condition was detected.

## Discussion

Information on single cases and a small case series of imported leishmaniases in Germany is available, but systematic reporting on frequency, type, and origin of leishmanial infections in Germany did not exist until 2000 (*5*–*7*). Our recent surveillance is dependent on passive consultation and reporting and therefore may have selection bias because if visceral leishmaniasis, a potentially fatal disease that requires hospitalization, is suspected, advice on diagnosis and treatment is sought more often than for the skin infection. We assume that our system captures approximately half of the visceral leishmaniasis cases and approximately one third of the classical cutaneous cases imported to Germany.

A total of 47% of all cases, but 78% of the visceral cases were contracted in the European Mediterranean area and Portugal, and most of the infections indicated a species of the *L. donovani* complex, most probably *L. infantum*, as the probable causative agent. Thirteen infections (22%) were acquired on the Mediterranean islands of Ibiza, Ischia, Majorca, Malta, Korfu, or Sicily.

This distribution reflects the fact, that the Mediterranean countries, Spain, Italy, and the Mediterranean islands, in particular, are the favorite vacation areas for Germans. Annually, Germans take 18 million vacations to the European Mediterranean area (including 8 million to Spain and 6 million to Italy) with a median duration of 2 weeks. Sixty percent of travel to Italy and 90% of travel to Spain are to *Leishmania*-endemic areas.

While leishmaniasis has always been endemic in the Mediterranean countries, the maximum northern latitude for sandfly survival is speculated to move further to the North, beyond Germany (*1*) because of global warming. If this scenario is correct, the imported cases may serve as a potential substrate for the sandfly vector. Dogs that are imported as pets from the disease-endemic areas of southwestern Europe or that contract the infection when accompanying their owners for vacation are another potential substrate (*8*).

Infections with *L. infantum* in a child, as well as in a horse who had never left Germany, have recently been described and have led to speculations about an autochthonous focus (*9*,*10*). Also recently, the first sandfly species, *Phlebotomus mascittii* Grassi, 1908, was detected in southern Germany, although its potential as a vector of *Leishmania* remains to be demonstrated (*11*).

As expected, visceral leishmaniasis is often manifested in persons with impaired immunocompetence because of young age, HIV infection, immunosuppressive therapy and, in our analysis, in older persons with concomitant diseases.

Notably, 12 (67%) of 18 of the visceral cases contracted in the European Mediterranean area were in adults, thus confirming a change in age groups affected. Formerly, visceral leishmaniasis was known mainly as a disease of children (*1*,*2*). This change may partly be explained by the increased proportion of *Leishmania* and HIV–co-infected persons and partly by increased travel activities of otherwise immunocompromised persons, including elderly persons.

Furthermore, even in patients with cutaneous leishmaniasis, dissemination of parasites has to be excluded in case of impaired immunocompetence (e.g., immunosuppressive treatment). In these cases, *Leishmania*-specific PCR of the buffy coat of the peripheral blood is a sensitive method for detecting parasite spread beyond the skin.

Parents of small children and persons with reduced immunocompetence should be informed about their increased susceptibility to infection with *Leishmania* when traveling to disease-endemic areas. Measures to reduce the exposure to sandflies, such as clothes, repellents, and mosquito nets as well as collars impregnated with repellents for accompanying dogs, should be recommended.
